# Efficacy and safety of apheresis therapy in AQP4 antibody‐positive NMOSD attack: A propensity score‐matched cohort study

**DOI:** 10.1111/cns.14780

**Published:** 2024-05-24

**Authors:** Yun Xu, Huabing Wang, Tian Song, Linlin Yin, Yajun Yao, Yuzhen Wei, Hengri Cong, Jiali Sun, Xinghu Zhang, De‐Cai Tian

**Affiliations:** ^1^ Center for Neurology, Beijing Tiantan Hospital Capital Medical University Beijing China; ^2^ China National Clinical Research Center for Neurological Diseases Beijing China

**Keywords:** acute attack, immunoadsorption, NMOSD, plasma exchange, PSM

## Abstract

**Objective:**

Plasma exchange (PE) and immunoadsorption (IA) are recognized as effective ways to treat attacks in AQP4 antibody‐positive NMOSD, but high‐quality evidence was lacking. To evaluate the efficacy and safety of PE/IA plus intravenous methylprednisolone (IVMP) in NMOSD attacks using propensity scores to match IVMP as control.

**Methods:**

Patients were from a prospective observational cohort study. Stratification and interval propensity score matching (PSM) were used to reduce selection bias by matching baseline characteristics (gender, age, time to IVMP, EDSS at attack) between PE/IA + IVMP and IVMP group (in a ratio of 1:2). The primary endpoint of efficacy was EDSS change at 6 months. Adverse events and changes in laboratory tests were recorded.

**Results:**

Four hundred and eleven attacks of 336 patients were screened for PSM, and 90 attacks (30 PE/IA + IVMP and 60 IVMP) were included in the analysis. There were significant differences in EDSS [6.25 vs. 6.75; IQR (4.50–8.38 vs. 5.00–8.00), *p* = 0.671] and visual acuity [median logMAR = 0.35 vs. 1.00; IQR (0.30–0.84 vs. 0.95–1.96), *p* = 0.002] change between two groups at 6 months. PE/IA + IVMP treatment demonstrated predictive capacity for good recovery as indicated by an area under the curve (AUC) of 0.726. Fibrinogen reduction was found during PE/IA + IVMP treatment [*n* = 15 (50.00%)], but no severe adverse events led to apheresis treatment discontinuation.

**Discussion:**

After PSM analysis, IVMP+PE/IA in acute attack of NMOSD achieved better and continuous improvement in neurological function within 6 months compared with IVMP alone. PE/IA treatment showed a good safety profile.

## Graphical abstract

After propensity score matching (PSM) analysis, IVMP+PE/IA in acute attack of NMOSD achieved better and continuous improvement in neurological function within 6 months compared with IVMP alone, especially optic neuritis. PE/IA treatment showed a good safety profile.

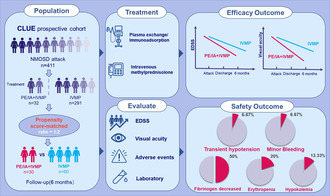

## 1 INTRODUCTION

Neuromyelitis optica spectrum disorder (NMOSD) is characterized by aquaporin‐4 antibody‐mediated astrocytepathy, which leads to blindness and paralysis after severe acute attack.^1^ Acute treatment is critically important as exacerbations result in severe residual disability.^2^ High‐dose intravenous methylprednisolone (IVMP) was initially essential in acute relapse,^3^ but remission of symptoms was in only 19% of patients.^4^ Moderate‐to‐severe relapses and those that respond incompletely to IVMP may be ameliorated with early administration of apheresis therapies.^5^

Apheresis therapies, which include the two main therapies plasma exchange (PE) and immunoadsorption (IA), aim to eliminate disease‐causing antibodies and other pro‐inflammatory factors from the patient's peripheral circulation.^6^, ^7^, ^8^ Although PE/IA + IVMP treatment was found to be effective in previous retrospective studies, high‐quality evidence was lacking.^9^, ^10^, ^11^, ^12^, ^13^

Randomized placebo‐controlled trials of PE/IA + IVMP treatment are necessary to provide high‐quality evidence. However, the randomized, placebo‐controlled trial of PE/IA in NMOSD patients with acute attack is absent. PSM is a powerful statistical technique for correcting multiple baseline covariate imbalances in nonrandomly selected cohorts, which minimizes the influence of selection bias.^14^, ^15^ The objective of this study was to provide high‐level evidence of PE/IA treatment via PSM in NMOSD acute attacks.

## 2 METHODS

### 2.1 Database and study population

The patients were from the clinical and imaging patterns of neuroinflammation diseases in China (CLUE, NCT04106830), an ongoing prospective cohort study among patients with demyelination disease in China. Data of patients with a diagnosis of NMOSD were extracted from the CLUE from September 2019 to July 2023. Inclusion criteria: (1) diagnosis meeting the 2015 Revised Criteria,^16^ (2) with serum AQP4‐IgG positive, (3) with acute attack (from September 2019 to February 2023), and (4) age ≥18 years old. Exclusion criteria: (1) other treatments except IVMP and PE/IA + IVMP were received in the acute phase, (2) relapse within 6 months after this acute attack, (3) initial Expanded Disability Status Scale (EDSS) score ≥5 before this acute attack, (4) IVMP delayed ≥3 weeks, (5) coexist other CNS inflammatory diseases, and (6) patients with heart, liver, lung, and renal failure and severe infections and malignant tumors. The CLUE cohort was pre‐set for imaging and clinical evaluation (EDSS and visual acuity) on the first day of admission and 6 months after discharge. For this analysis, the required dataset consisted of sex, birth, date of first NMO symptoms, dates of clinical relapses, clinical NMO course, and disability quantified with EDSS and visual acuity recorded at the time of attack, discharge, and 6 months.

Visual acuity values were assessed by Sloan 2.5% low contrast visual acuity chart and were changed into LogMAR values for analysis. TheLogMAR value for count fingers (CF) is 1.85, hand movement (HM) is 2.30, light perception (LP) is 2.70, and no light perception (NLP) is 3.00.^17^

Written informed consent was provided by all participants or their guardians. This project was approved by the Ethics committee of Beijing Tiantan Hospital, Capital Medical University, Beijing, China (KY 2019‐050‐02).

### 2.2 Treatment

All attacks were treated with intravenous methylprednisolone (IVMP) at a dose of 500 mg/day for three consecutive days, followed by 240 mg/day for three consecutive days, then 120 mg/day for three consecutive days, and a taper of oral prednisolone.

PE/IA was implanted if limited response or deterioration judged by the physician in neurologic function (strength or sensation of limb, visual acuity, or bowel/bladder function) after IVMP treatment (methylprednisolone was reduced to 120 mg). During IA/PE, vital signs (heart rate, blood pressure, and oxygen saturation) are monitored continuously in all patients. The plasma exchange was performed after the placement of a Shaldon catheter in the jugular vein. PE was performed on a continuous flow filtration device (Prismaflex) with a Shaldon catheter every other day for a total of five sessions; one volume of plasma was exchanged against 5% human albumin solution. Internal jugular veins were used for central vascular access with double‐lumen catheters in all patients. Anticoagulation was done with low‐molecular‐weight heparin. IA was performed using the tryptophan‐linked polyvinyl alcohol adsorber TR‐350 (Asahi Kasei Medical, Tokyo, Japan) treating one plasma volume with a maximum of 3000 mL plasma per session for all treatments by jugular central venous catheters. Five sessions were performed within 10–12 days, and treatment‐free intervals were extended for complications or decrease of fibrinogen below 100 mg/dL.

### 2.3 Outcome measure definition

The EDSS was evaluated at baseline, at attack, at discharge, and at 6 months. We analyzed the change in EDSS during follow‐up as the primary outcome of efficacy. The secondary outcome of efficacy was walking without restriction at 6 months, which is the disability status of a confirmed EDSS score of 4.5 or less; which corresponds to the ability to walk independently at least 500 m without resting. Safety endpoints included adverse events documented in the medical reports and the laboratory parameters. Values for safety laboratory parameters were appropriate normal ranges.

### 2.4 Propensity score matching

The included patients were matched on their propensity for receiving PE/IA or IVMP using the MatchIt package by R (v4.2.0). Stratification was performed according to the clinical attack phenotype (optic neuritis and other clinical phenotypes), and then propensity score matching was performed within intervals. Covariates were chosen for PSM based on two criteria: (1) being unrelated to the exposure (PE/IA treatment) and (2) being known to affect the risk of the outcome.^18^ Based on current evidence, the covariates included gender, age, time to IVMP, EDSS at attack/LogMAR value at attack, disease duration, and previous treatments.^19^, ^20^, ^21^, ^22^, ^23^ The incorporation of an excessive number of covariates may lead to a reduction in the quantity of suitable matches and a decrease in precision.^24^ Based on the results of Spearman's rank correlation analysis and the results of a balance test that compared the distribution of confounders in the matched samples, only four baseline covariates (gender, age, time to IVMP, EDSS at attack/LogMAR value at attack) were selected for PSM. We used a logistic regression model to calculate propensity score with treatment allocation as the outcome variable, the baseline characteristics (gender, age, time to IVMP, EDSS at attack/LogMAR value at attack) formed the independent variables. The matching technique used the nearest neighbors method within a caliper of 0.2, which allowed us to successfully match 1:2 each of the PE/IA + IVMP group to the IVMP group.

### 2.5 AQP4‐Ab testing

The test of serum AQP4 antibodies was carried out by NEW TERRAIN (Tianjin, China) or Kingmed Diagnostics (Guangzhou, China) before IVMP treatment.

### 2.6 Statistical analysis

Statistical analysis was conducted using SPSS 25.0 (International Business Machines Corporation, Chicago, IL, USA). Continuous variables were presented using mean and standard deviation (SD), or median and interquartile range (IQR). Categorical variables were summarized by frequency and percentage. Wilcoxon Signed Ranks Test and McNemar's tests were used to compare baseline characteristics in the matched data for continuous variables and proportions, respectively. A multivariable Cox analysis was run to identify 6‐month factors associated with the risk of reaching the primary outcome. The secondary outcome (changes of EDSS/mRS) analysis by Wilcoxon Signed Ranks Test. Model prediction performance of PE/IA + IVMP versus IVMP alone in EDSS improvement was assessed by the area under the receiver‐operating characteristic (ROC) curve (which is equivalent to the c statistic). Adverse events were analyzed descriptively. All two‐tailed values of *p* < 0.05 were considered significant.

## 3 RESULTS

### 3.1 Characteristics of participants and matching

**FIGURE 1:**
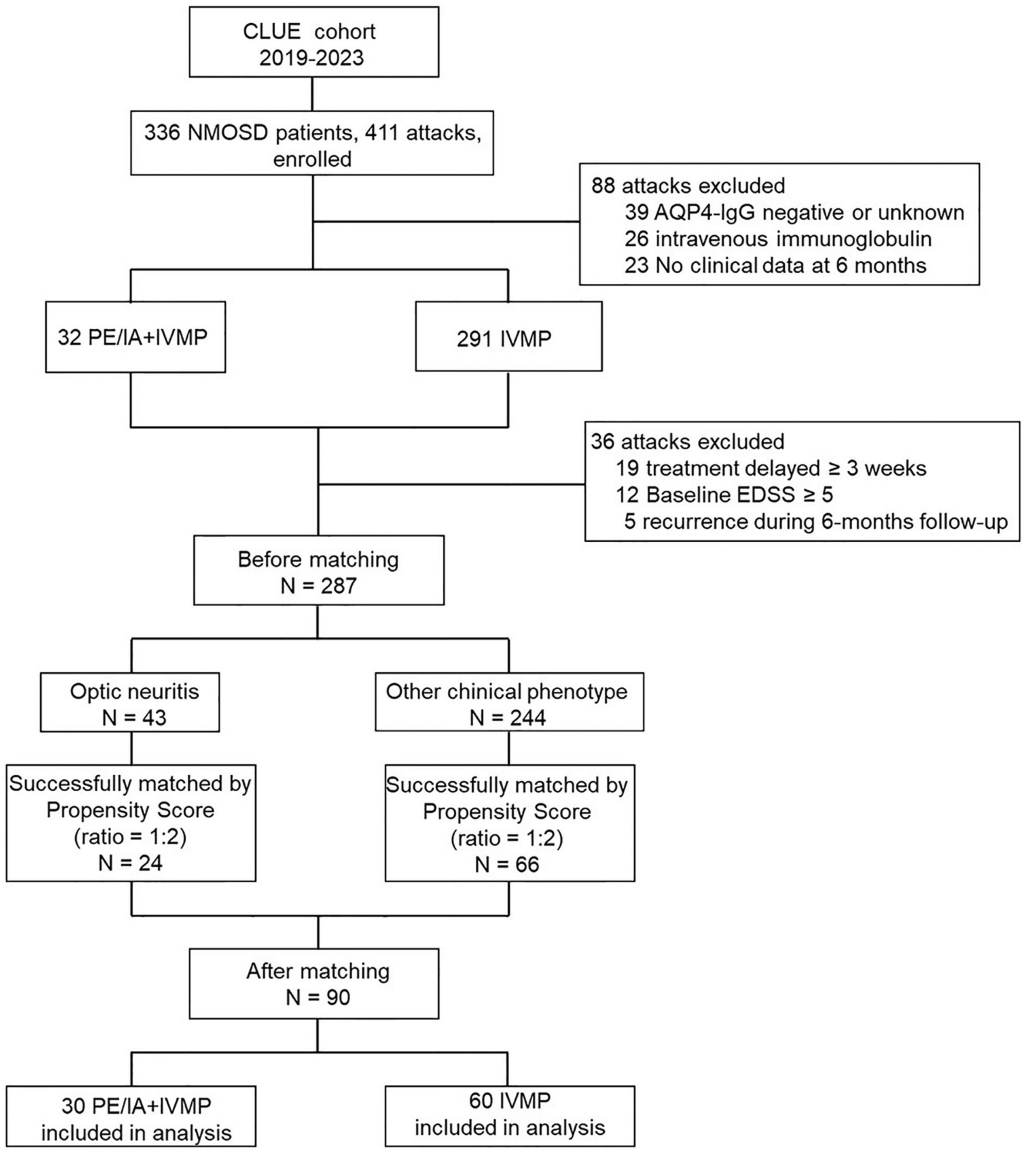
Study flowchart of patient disposition. EDSS, Expanded Disability Status Scale; IA, immunoadsorption; IVMP, intravenous methylprednisolone; NMOSD, neuromyelitis optica spectrum disorder; PE, plasma exchange.

Four hundred and eleven potentially eligible attacks (336 NMOSD patients) from the CLUE cohort were screened, of which 88 did not meet the inclusion criteria and were excluded. The remaining 323 attacks were enrolled, 36 of which were excluded during follow‐up for treatment delayed ≥3 weeks, Baseline EDSS ≥5, and recurrence. Of the included 287 attacks, 30 PE/IA + IVMP treatments could be matched (1:2) to an IVMP control (Figure 1).

Baseline demographics and key characteristics of the PE/IA + IVMP group (*n* = 30) and propensity score‐matched IVMP group (*n* = 60) are shown in Table 1. The two groups (PE/IA + IVMP and IVMP group) were successfully matched by age, gender, delay from onset to treatment, and EDSS at attack. The matching procedure significantly improved the overall match (Tables S1 and S2) and improved the distributional balance for “distance” (Figure S1). In each group, BMI, disease duration, annual relapse rate, attack times, and coexisting autoimmune diseases were not statistically different. No significant differences in the proportion of first attack were found between the two groups (PE/IA + IVMP vs. IVMP) [8 (26.67%) vs. 24 (40.00%), *p* = 0.213]. Few patients were given relapse prevention treatment in two groups [9 (30.0%) vs. 12 (20.0%), *p* = 0.310], including Rituximab, Mycophenolate Mofetil, and Azathioprine. Median baseline EDSS scores (before attack) in PE/IA + IVMP group were 0 (IQR, 0–2.88) and 0 (IQR, 0–0.5) in IVMP group (*p* = 0.509). EDSS scores at attack were 4.5 (IQR, 4.00–7.75) in PE/IA + IVMP group and 5.75 (IQR, 4.00–7.62) in IVMP group (*p* = 0.841). Visual acuity at attack was 1.85 (IQR, 1.00–2.06) in PE/IA + IVMP group and 2.08 (IQR, 0.95–2.70) in IVMP group (*p* = 0.853). The serum AQP4‐IgG antibody titers of the PE/IA + IVMP group at attack were similar to IVMP group [median titers 1:100; IQR (1:32–1:100), *p* = 0.727].

**TABLE 1:** Baseline characteristics of the included NMOSD patients after matching.

	PE/IA + IVMP (*n* = 30)	IVMP (*n* = 60)	*p*
Age, mean (SD), years	43.90 (13.98)	41.55 (15.85)	0.493
Female, *n* (%)	27 (90.00)	54 (90.00)	1.000
BMI (kg/m^2^), median (IQR)	22.87 (21.48–24.99)	21.48 (19.53–24.08)	0.055
Disease duration, median (IQR), years	1.50 (0.50–4.38)	0.42 (0.17–4.00)	0.071
Annual relapse rate, mean (SD)	1.03 (1.57)	1.15 (2.04)	0.789
First attack, *n* (%)	8 (26.67)	24 (40.00)	0.213
Second attack, *n* (%)	6 (20.00)	17 (28.33)	0.632
≥3 attacks, *n* (%)	16 (53.33)	18 (30.00)	0.031*
Delay from onset to IVMP, median (IQR), days	11.50 (7.00–16.50)	10.50 (6.00–15.00)	0.810
Delay from onset to IA/PLEX, median (IQR), days	17.5 (15.00–25.00)	NA	NA
Clinical manifestation, *n* (%)			
Optic neuritis	8 (26.67)	16 (26.67)	1.000
LETM	20 (66.67)	40 (66.67)	
Brain	1 (3.33)	1 (1.67)	
Brainstem	1 (3.23)	3 (5.00)	
Coexisting autoimmune diseases, *n* (%)	14 (46.67)	21 (35.00)	0.285
Relapse prevention treatment after attack, *n* (%)			
RTX	2 (6.67)	2 (3.33)	0.310
MMF	6 (20.00)	5 (8.33)	
AZA	0 (0.00)	3 (5.00)	
Other	1 (3.33)	2 (3.33)	
Not use	21 (70.00)	48 (80.00)	
Baseline EDSS, median (IQR)	0 (0.00–2.88)	0 (0.00–0.50)	0.509
Attack EDSS, median (IQR)	4.50 (4.00–7.75)	5.75 (4.00–7.62)	0.841
Attack visual acuity, logMAR, median (IQR)	1.85 (1.00–2.06)	2.08 (0.95–2.70)	0.853
Serum AQP4‐IgG titer at attack, median (IQR)	1:100 (1:32–1:100)	1:100 (1:32–1:100)	0.727

Abbreviations: AZA, azathioprine; BMI, body mass index; EDSS, Expanded Disability Status Scale (*p < 0.5); IA, immunoadsorption; IQR, inter‐quartile range; IVMP, intravenous methylprednisolone therapy; LETM, longitudinal extensive transverse myelitis; MMF, mycophenolate mofetil; NMOSD, neuromyelitis optica spectrum disorder; PE, plasma exchange; RTX, rituximab; SD, standard deviation.

### 3.2 Effects of PE/IA therapy

**FIGURE 2:**
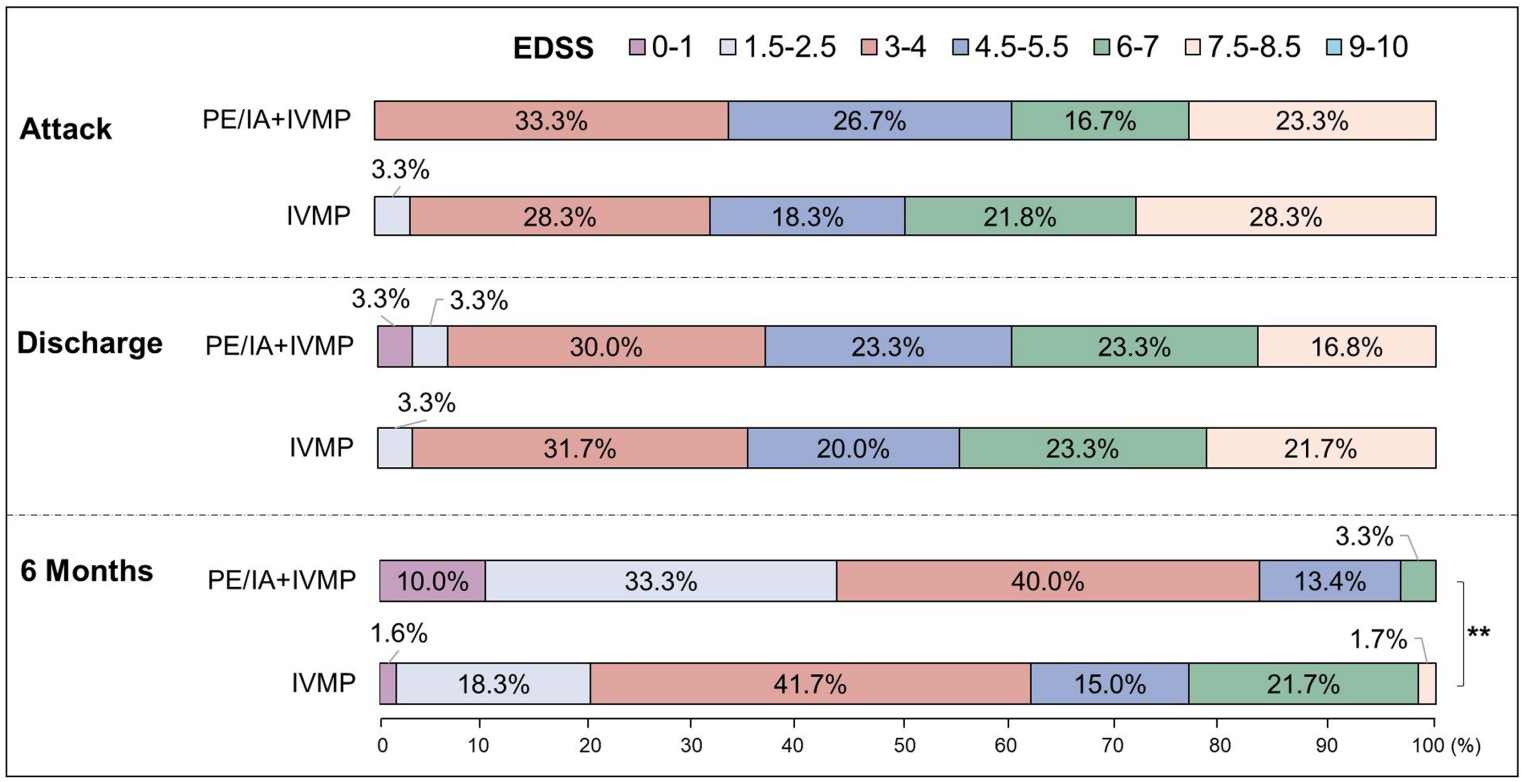
Distribution of EDSS scores at attack, at discharge, and 6 months in two groups (**p < 0.01).

EDSS score changes of PE/IA + IVMP and IVMP groups are shown in Figure 2. There was no difference in the distribution of EDSS between PE/IA + IVMP and IVMP groups at an attack [odds ratio (OR), 0.915; 95% confidence interval (CI), 0.639–1.309; *p* = 0.627]. Although, there was no significant difference between the two groups in the distribution of EDSS at discharge (OR 0.887; 95% CI 0.621–1.266; *p* = 0.508). A more favorable shift of EDSS distribution (less disability) was found in PE/IA + IVMP group compared to IVMP group at 6 months (OR 0.499; 95% CI 0.309–0.806; *p* = 0.004).

**FIGURE 3:**
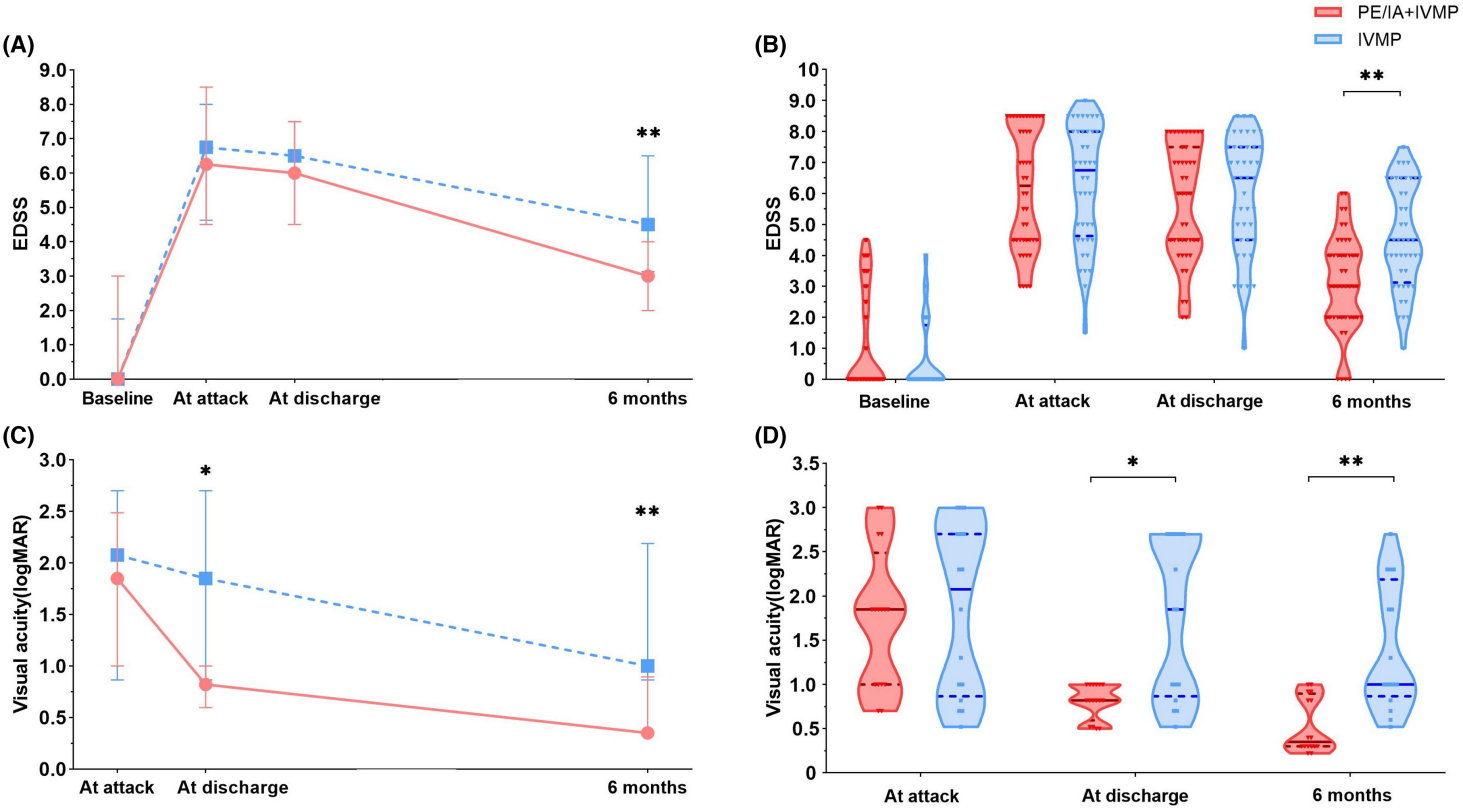
The changes in EDSS and visual acuity in PE/IA and IVMP groups after follow‐up. (A) The changing trend of EDSS score within 6 months. (B) EDSS score distribution in PE/IA group and IVMP group within 6 months. (C) Visual acuity changes before and after treatment. (D) Visual acuity distribution in PE/IA group and IVMP group within 6 months. *p < 0.05, **p < 0.01.

At attack, median EDSS in PE/IA + IVMP and IVMP groups (other phenotypes except optic neuritis) was similar [median EDSS 6.25 vs. 6.75; IQR (4.50–8.38 vs. 5.00–8.00), *p* = 0.671]. PE/IA + IVMP and IVMP groups showed improvement with EDSS but no difference between the two groups at discharge [median EDSS 6.00 vs. 6.50; IQR (4.50–7.38 vs. 5.00–7.50), *p* = 0.432]. At 6‐months, the EDSS of both groups had significantly improved, but the EDSS improvement of the PE/IA + IVMP group was better than that of the IVMP group [median EDSS 3.00 vs. 4.50; IQR (2.00–4.00 vs. 3.00–6.50), *p* = 0.003] (Figure 3).

Visual acuity had no significant difference at attack between the two groups (optic neuritis) [median logMAR = 1.85 vs. 2.08; IQR (1.00–2.06) vs. (0.95–2.70), *p* = 0.853]. Visual acuity improved in PE/IA + IVMP higher than that in IVMP groups at discharge [median logMAR = 0.82 vs. 1.85; IQR (0.74–1.00) vs. (0.95–2.70), *p* = 0.025]. At 6 months, PE/IA + IVMP did have significantly lower logMAR than IVMP group [median logMAR = 0.35 vs. 1.00; IQR (0.30–0.84 vs. 0.95–1.96), *p* = 0.002] (Figure 3). We conducted a subgroup analysis of patients with different number of attacks, and found that PE/IA + IVMP therapy shows a more pronounced effect in patients with first attacks (Figures S2–S4).

### 3.3 Predictors of prognosis

**FIGURE 4:**
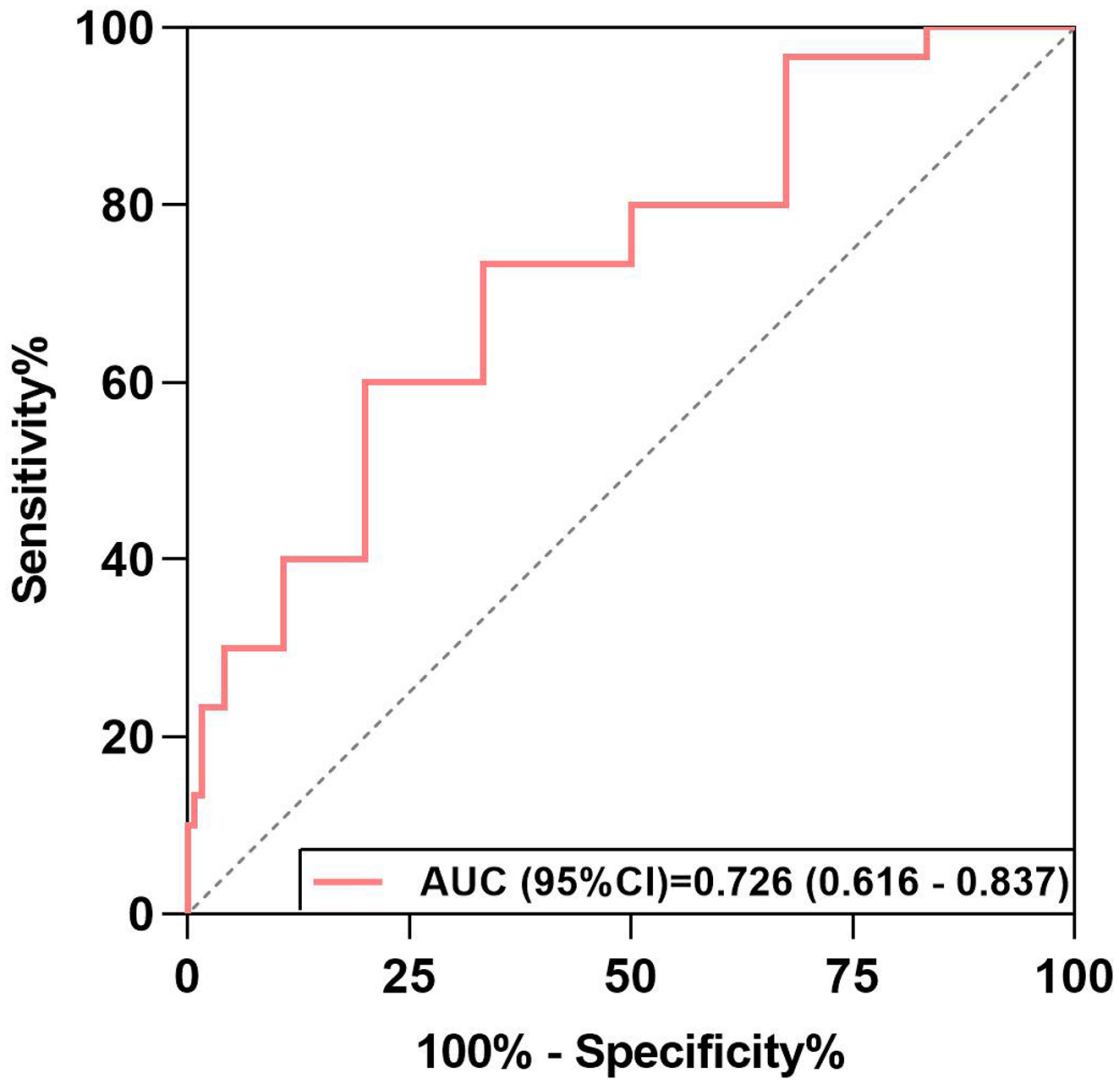
ROC curves for prognosis predictive value of PE/IA + IVMP treatment.

We performed an analysis of seven potentially valuable independent predictors of outcome (Table 2). In univariate analysis in IVMP group, improvement was predicted by age (OR 0.959; 95% CI 0.925–0.994; *p* = 0.022), BMI (OR 0.826; 95% CI 0.692–0.986; *p* = 0.035), EDSS at attack (OR 0.229; 95% CI 0.114–0.458; *p* < 0.001), serum AQP4‐IgG titer at attack (OR 0.996; 95% CI 0.992–1.000; *p* = 0.047), whereas annual relapse rate (OR 0.963; 95% CI 0.747–1.240; *p* = 0.768), attack times (OR 0.870; 95% CI 0.605–1.252; *p* = 0.454), and delay from onset to IVMP (OR 1.056; 95% CI 0.962–1.158; *p* = 0.252) demonstrated no significant influence. In multivariate analysis, improvement was influenced independently by EDSS at attack (OR 0.181; 95% CI 0.074–0.441; *p* < 0.001). ROC curve was used for the prognosis predictive value of PE/IA + IVMP treatment, the area under the ROC curve (Figure 4) was 0.726 (0.616–0.837).

**TABLE 2:** Univariate and multivariate logistic regression analysis of prognosis predicts factors in NMOSD attack (IVMP alone).

	Univariate analysis			Multivariate analysis		
	OR	95% CI	*p*	OR	95% CI	*p*
Age	0.959	0.925–0.994	0.022*	1.064	0.979–1.156	0.142
BMI, median, kg/m^2^	0.826	0.692–0.986	0.035*	0.727	0.488–1.083	0.083
Annual relapse rate	0.963	0.747–1.240	0.768	—	—	—
Attack times	0.870	0.605–1.252	0.454	—	—	—
Delay from onset to IVMP	1.056	0.962–1.158	0.252	—	—	—
EDSS at attack	0.229	0.114–0.458	<0.001**	0.181	0.074–0.441	<0.001**
Serum AQP4‐IgG titer at attack	0.996	0.992–1.000	0.047*	0.997	0.992–1.002	0.234

Abbreviations: CI, confidence interval; NMOSD, neuromyelitis optica spectrum disorder; OR, odds ratio.

*p < 0.05, **p < 0.01 represents statistical significance.

### 3.4 Side effects and laboratory tests under PE/IA therapy

Of all treated patients, 86.67% (26 of 30 attacks) were free of any adverse event (Table 3). In total, four adverse reactions in 30 apheresis treatments (13.33%) were documented, two transient hypotension during apheresis treatments, one nosebleed, and one bleeding at the catheter site during IA + IVMP. Laboratory index revealed fibrinogen decreased, thrombocytopenia, erythropenia, and hypokalemia as the common side effects. Patients with fibrinogen decreased (50.00%) and thrombocytopenia (6.67%) were all from IA + IVMP group. Erythropenia (25.00% vs. 18.18%) occurred in the IA + IVMP and the PE group. Hypokalemia was more frequent in PE + IVMP than IA + IVMP group (25.00% vs. 9.09%). No leukopenia was observed in both groups. No patient stopped apheresis treatment due to side effects.

**TABLE 3:** Adverse events and laboratory index under PE/IA + IVMP therapy.

	Total (*n* = 30)	Treatment		*p*
		PE + IVMP (*n* = 8)	IA + IVMP (*n* = 22)	
Adverse events				
Transient hypotension, *n* (%)	2 (6.67)	1 (12.50)	1 (4.55)	0.469
Hematoma/bleeding, *n* (%)	2 (6.67)	0	2 (9.09)	1.000
Nausea/vomitus, *n* (%)	0	0	0	—
Allergic reaction, *n* (%)	0	0	0	—
Thrombosis, *n* (%)	0	0	0	—
Arrhythmia, *n* (%)	0	0	0	—
Air‐embolism, *n* (%)	0	0	0	—
Laboratory index				
Fibrinogen decreased, *n* (%)	15 (50.00)	0	15 (68.18)	0.002**
Leukopenia, *n* (%)	1 (3.33)	0	1 (4.55)	1.000
Thrombocytopenia, *n* (%)	2 (6.67)	0	2 (9.09)	1.000
Erythropenia, *n* (%)	6 (20.00)	2 (25.00)	4 (18.18)	0.645
Hypokalemia, *n* (%)	4 (13.33)	2 (25.00)	2 (9.09)	0.284

Abbreviations: IA, immunoadsorption; IVMP, intravenous methylprednisolone therapy; PE, plasma exchange.

**p < 0.01

## 4 DISCUSSION

In this real‐world cohort study, we explore the effectivity and safety of apheresis therapy in AQP4 antibody‐positive NMOSD via propensity score matching to achieve pseudo‐randomization. IVMP combined with PE/IA achieved better and continuous improvement than IVMP alone; PE and IA were safe and no life‐threatening complications were found in acute attack treatment in NMOSD.

Using the PSM method, we found that patients improved significantly after apheresis therapy while reducing the selection bias.^25^ Randomized, blinded studies are an ideal method for comparing two different treatments for any disease. Currently, there is no prospective cohort study on apheresis therapy in NMOSD patients, but several retrospective studies have shown the same conclusion.^5^, ^9^, ^26^, ^27^, ^28^ It is difficult to avoid selection bias in retrospective studies, and patients receiving PE/IA + IVMP often have more severe clinical symptoms and poor response to IVMP alone.^13^, ^27^ We matched the gender, age, time to treatment, and EDSS at attack of the two groups to reduce the impact of these factors on the analysis of treatment outcomes. Although the two groups of patients did not show significant differences in EDSS at discharge and 1 month, it was found that the PE/IA + IVMP treatment group achieved a sustained improvement at 6 months – PE/IA + IVMP may have a more durable effect on acute attack. This may be related to the mechanism of PE/IA: quickly clear serum AQP4‐IgG and complement, reduce the damage of astrocytes caused by membrane attack complex; and regulate lymphocyte subsets and cytokines to reduce disease activity.^7^

We found prognostic factors after acute episodes of NMOSD included: receiving corticosteroids combined with PE/IA was a predictor of a good outcome, whereas severe acute disability (high EDSS score) was a predictor of a poor outcome. Prognostic factors for apheresis therapy response have also been reported in previous studies, including shorter disease duration, reflex reserved, early initiation of apheresis therapy, early improvement, lower baseline EDSS scores, fewer past relapses, and non‐ON attack.^26^, ^29^, ^30^, ^31^ Therefore, patients with severe attacks and poor IVMP response should initial PE/IA treatment.

Compared with the application of PE/IA + IVMP to other attacks of NMOSD, the application in the ON attack is more efficient. In this study, the visual acuity of eight patients treated with IA/PE + IVMP had more pronounced and earlier (at discharge) improvement. This may be related to the role of PE/IA in clearing pathogenic antibodies, complement, and immune regulation to reduce the damage to the optic nerve in the early stage.^8^, ^32^

PE and IA constitute safety therapeutic options in NMOSD acute attacks. We recorded two patients (6.45%) in the PE group with transient hypotension, which recovered quickly after rehydration therapy. Previous studies have found that patients with neuroinflammatory diseases receiving PE have a higher risk of hypotension than those with internal diseases, possibly due to the higher incidence of autonomic dysfunction associated with neuroinflammatory diseases.^33^ Therefore, more frequent monitoring of blood pressure, reduction of blood flow rates, and plasma replacement rates in patients with NMOSD may be warranted. Minor bleeding (epistaxis and bleeding at the catheter site) occurred in two patients (9.09%) and fibrinogen decrease occurred in 15 patients (48.39%) but recovered quickly after pressure hemostasis and fibrinogen infusion. This finding is consistent with previous studies that monitoring fibrinogen levels during treatment and maintaining high fibrinogen levels during PE may help improve safety.^34^, ^35^, ^36^

Our study has several limitations as follows:(1) This is a real‐world prospective cohort rather than randomized clinical trials, but propensity matching techniques have maximized the reliability of the evidence; (2) In our clinical center, PE/IA was only used as rescue therapy in our daily practice (initiated when methylprednisolone was reduced to 120 mg), which may result in no significant different of outcome parameters between PE/IA + IVMP and IVMP groups at discharge^5^; (3) This is still a small sample study, and we look forward to the development of RCT related to PE/IA treatment of NMOSD in the future; (4) This study was conducted in an urban specialty tertiary medical center, which may have potential residual confounders related to healthcare access and demographics, and future multicenter studies may reduce this bias.

## 5 CONCLUSIONS

Under propensity matching analysis, apheresis therapy is effective in improving neurological function among the majority of patients with severe attacks, especially optic neuritis. PE/IA + IVMP treatments are relatively safe, with few and mild side effects.

## AUTHOR CONTRIBUTIONS

D.‐C.T and X.Z formulated the conception and design of this study. Y.Y, Y.W., and J.S. contributed to acquisition and analysis of clinical data. L.Y. contributed to assessing the AQP4‐Ab test results. H.C. and T.S. contributed to recurrence assessment. H.W. and L.Y. revised the manuscript. Y.X drafted the manuscript and prepared the figures.

## FUNDING INFORMATION

This work was supported by grants from the National Natural Science Foundation of China (82271374) and Beijing Natural Science Foundation grant (JQ23027).

## CONFLICT OF INTEREST STATEMENT

None declared.

## PROVENANCE AND PEER REVIEW

Not commissioned; externally peer‐reviewed.

## Supporting information


Appendix S1

## Data Availability

Anonymized data unpublished within this article are available from the corresponding author (Beijing Tiantan Hospital, Capital Medical University, Beijing, China) on reasonable request from any qualified investigator.
