# Supplementary material for: Efficacy and safety of apheresis therapy in AQP4 antibody‐positive NMOSD attack: A propensity score‐matched cohort study
Source: CNS Neurosci Ther. 2024 May 24;30(5):e14780. doi: 10.1111/cns.14780 (PMC11126786; doi:10.1111/cns.14780)
Supplement: Supplementary file 1 — Appendix S1 [file CNS-30-e14780-s001.docx]

**Supplementary Material**

1. **Supplementary Figures**
2. Supplementary Figure 1. Distributional balance of covariate before and after PSM
3. Supplementary Figure 2. Subgroup analysis of the changes in EDSS and Visual acuity in PE/IA and IVMP groups after follow-up (Subgroup of patients with first attack)
4. Supplementary Figure 3. Subgroup analysis of the changes in EDSS and Visual acuity in PE/IA and IVMP groups after follow-up (Subgroup of patients with second attack)
5. Supplementary Figure 4. Subgroup analysis of the changes in EDSS and Visual acuity in PE/IA and IVMP groups after follow-up (Subgroup of patients ≥ 3 attack)
6. Supplementary Figure 5. The association between serum AQP4-IgG titer and EDSS was analysed by Spearman’s correlation (PE/IA+IVMP group)
7. **Supplementary Tables**
8. Supplement Table 1. Baseline Characteristics of the Optic neuritis Groups Before and After 1:2 Matching for Predictive Variables
9. Supplement Table 2. Baseline Characteristics of other Clinical phenotype (except Optic neuritis) Groups Before and After 1:2 Matching for Predictive Variables
10. Supplement Table 3. Univariate regression analysis of AQP4-IgG titers at attack in EDSS improvement

**Supplementary Figures**

**
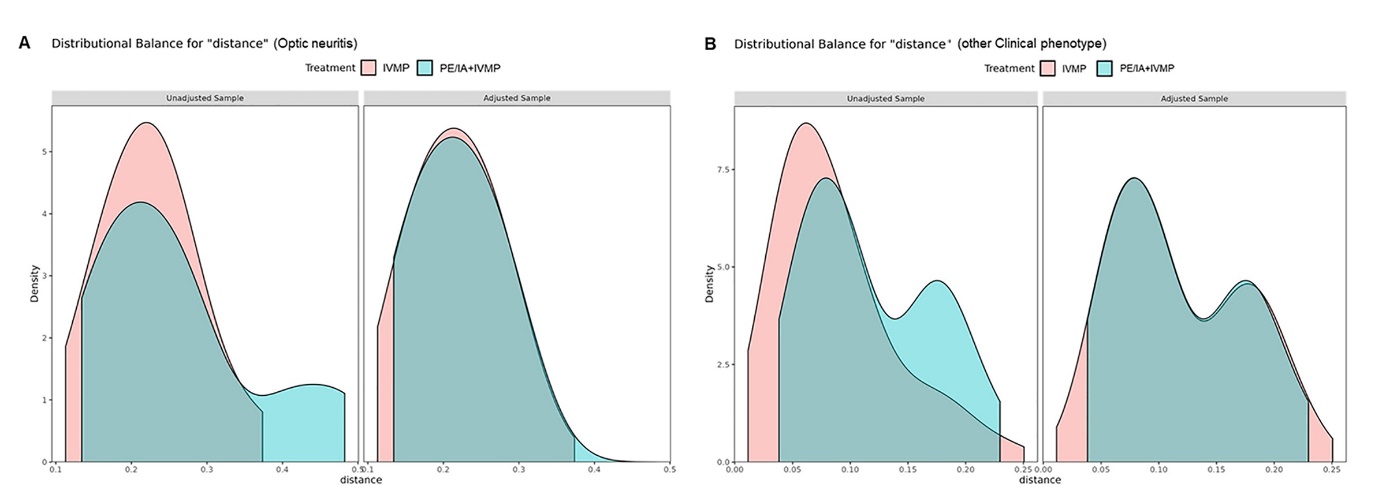
**

**Supplementary Figure 1. Distributional balance of covariate before and after PSM.**

**(A)** Distributional balance of covariate before and after PSM in optic neuritis group. **(B)** Distributional balance of covariate before and after PSM in other clinical phenotype group.

**
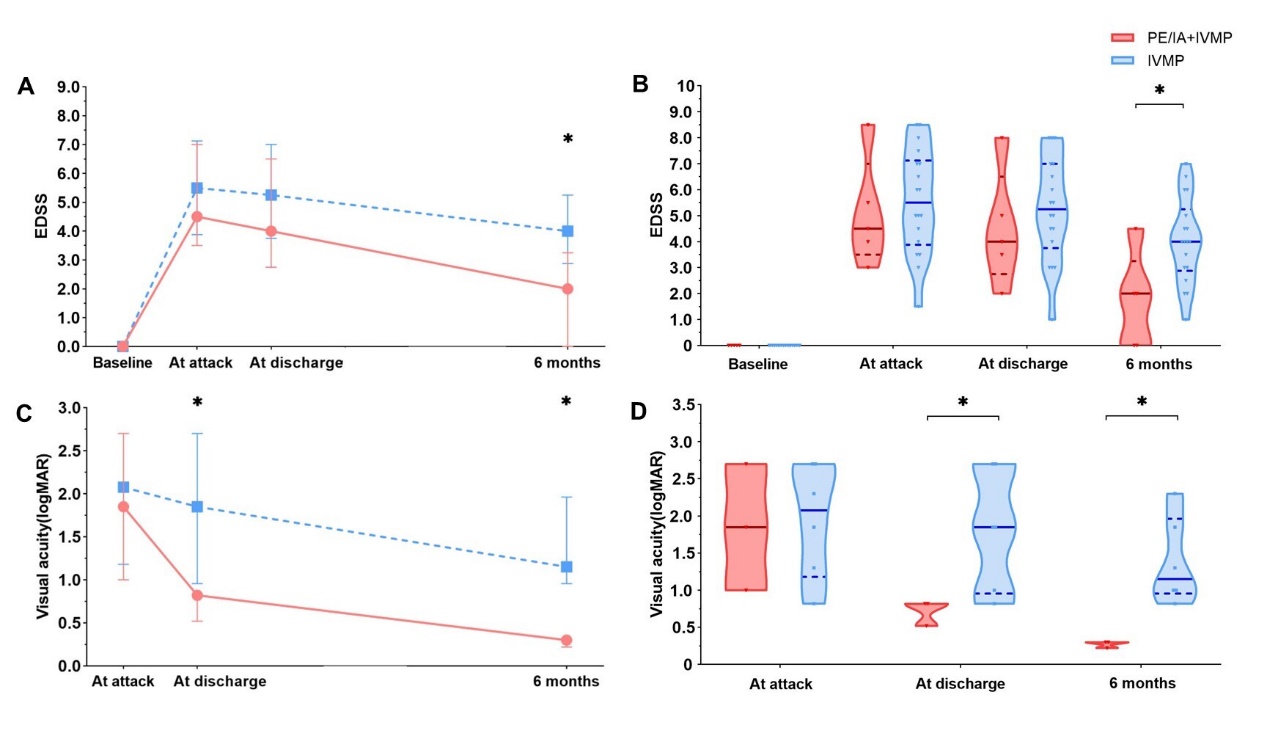
**

**Supplementary Figure 2. Subgroup analysis of the changes in EDSS and Visual acuity in PE/IA and IVMP groups after follow-up (Subgroup of patients with first attack, * P ＜0.05 ).**

**(A)** The changing trend of EDSS score within 6 months. **(B)** EDSS score distribution in PE/IA group and IVMP group within 6 months. **(C)** Visual acuity changes before and after treatment. **(D)** Visual acuity distribution in PE/IA group and IVMP group within 6 months. * P ＜0.05.


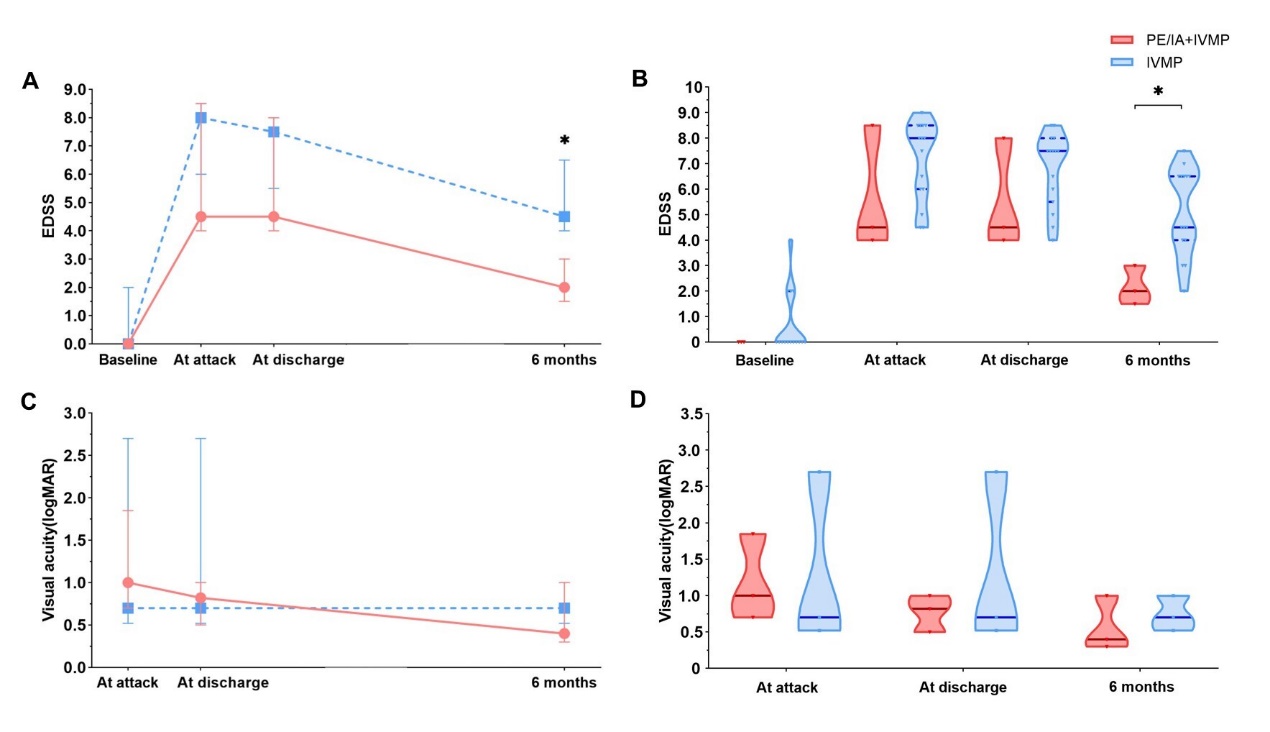


**Supplementary Figure 3. Subgroup analysis of the changes in EDSS and Visual acuity in PE/IA and IVMP groups after follow-up (Subgroup of patients with second attack, * P ＜0.05 ).**

**(A)** The changing trend of EDSS score within 6 months. **(B)** EDSS score distribution in PE/IA group and IVMP group within 6 months. **(C)** Visual acuity changes before and after treatment. **(D)** Visual acuity distribution in PE/IA group and IVMP group within 6 months. * P ＜0.05.

**
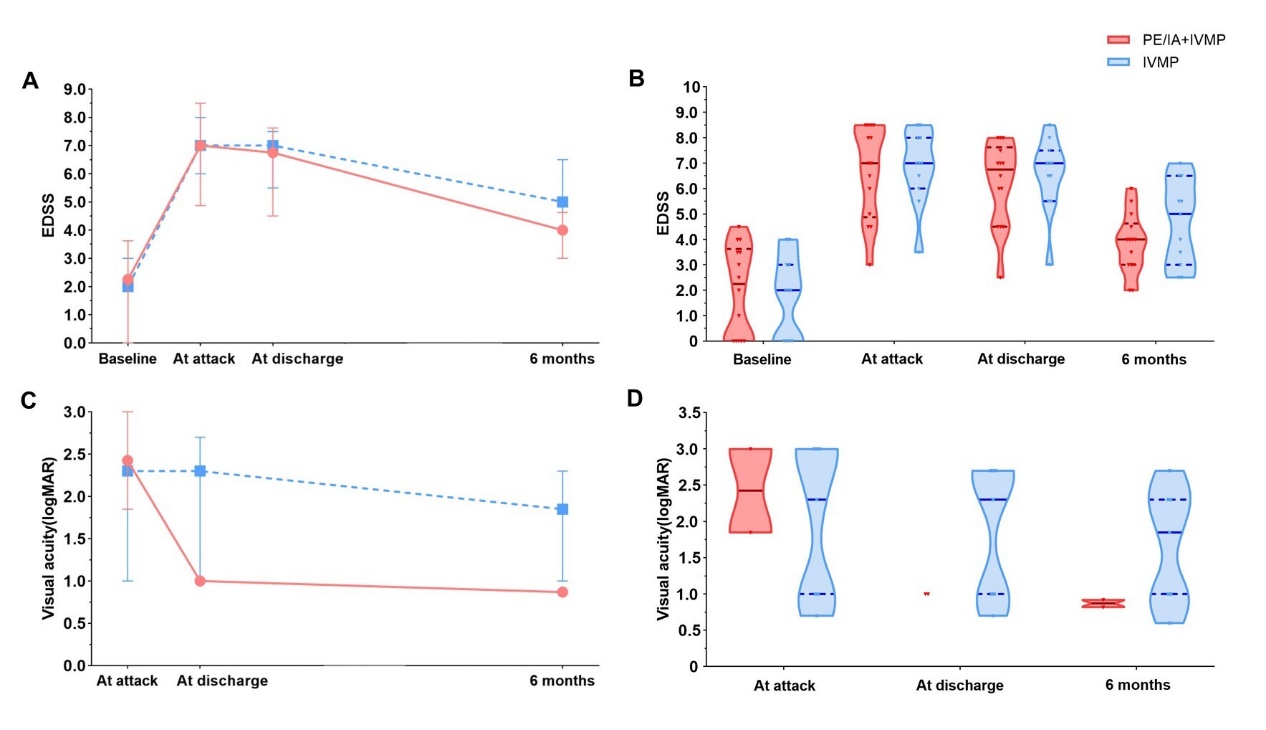
**

**Supplementary Figure 4. Subgroup analysis of the changes in EDSS and Visual acuity in PE/IA and IVMP groups after follow-up (Subgroup of patients ≥ 3 attacks, * P ＜0.05 ).**

**(A)** The changing trend of EDSS score within 6 months. **(B)** EDSS score distribution in PE/IA group and IVMP group within 6 months. **(C)** Visual acuity changes before and after treatment. **(D)** Visual acuity distribution in PE/IA group and IVMP group within 6 months. * P ＜0.05.


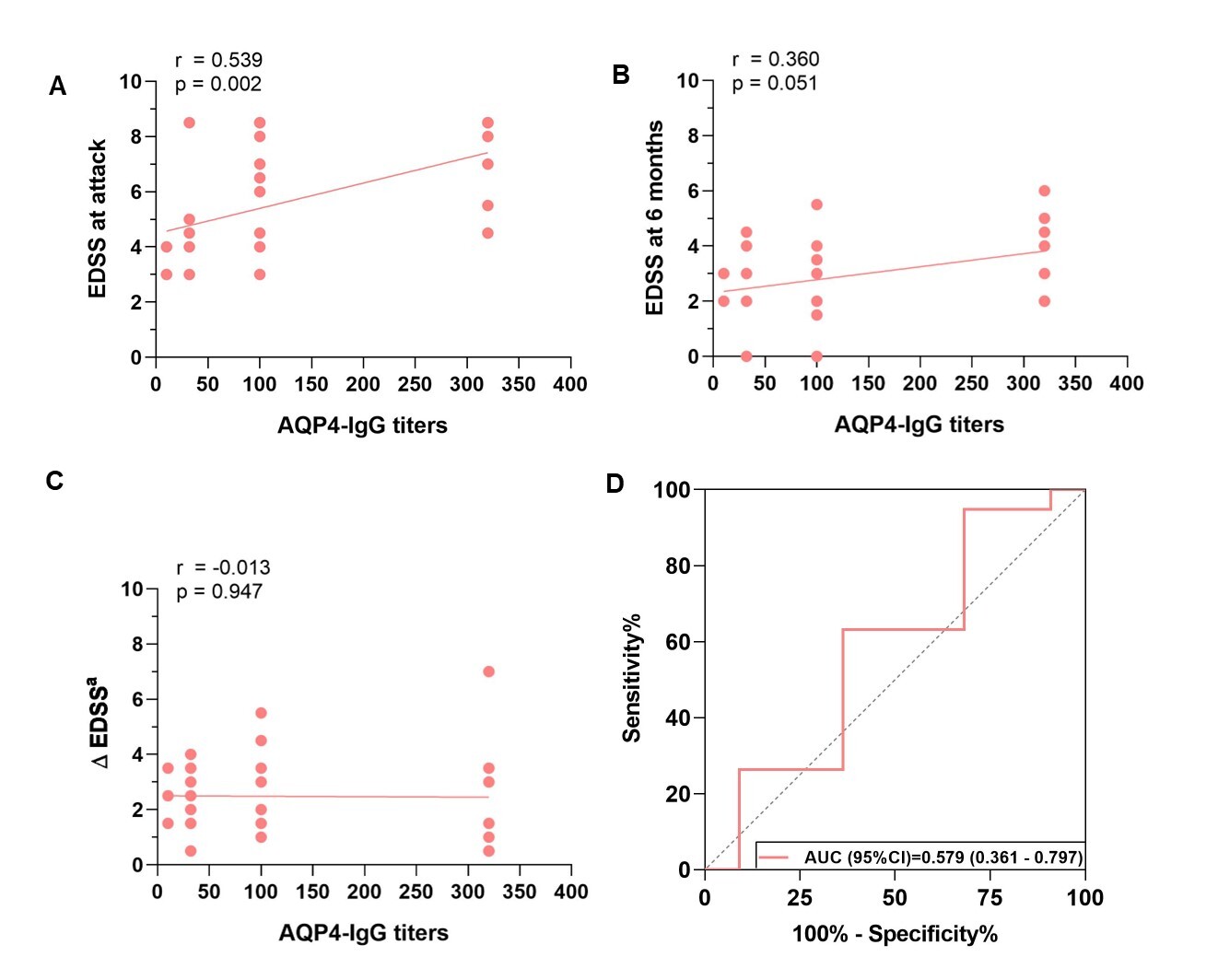


**Revised Supplementary Figure 5. The association between serum AQP4-IgG titer and EDSS was analysed by Spearman’s correlation (PE/IA+IVMP group).**

**(A)** The correlation between serum AQP4-IgG titer and EDSS at attack. **(B)** The correlation between serum AQP4-IgG titer and EDSS at 6 months. **(C)** The correlation between serum AQP4-IgG titer and ∆EDSS. **(D)** ROC curves for prognosis predictive value of serum AQP4-IgG titer at attack.

**Supplementary Tables**

| **Supplement Table 1.** Baseline Characteristics of the Optic neuritis Groups Before and After 1:2 Matching for Predictive Variables. | | | | | | | | | | |
| --- | --- | --- | --- | --- | --- | --- | --- | --- | --- | --- |
|  | **Before PSM** | | | |  |  | **After PSM** | | | |
|  | **Total**  **(n = 43)** | **IVMP**  **(n = 33)** | **PE/IA+IVMP**  **(n = 10)** | **P** |  |  | **Total**  **(n = 24)** | **IVMP**  **(n = 16)** | **PE/IA+IVMP**  **(n = 8)** | **P** |
| Age, mean (SD), years | 38.26 (11.83) | 37.30 (10.61) | 41.40 (15.47) | 0.344 |  |  | 34.79 (10.16) | 34.25 (9.76) | 35.88 (11.54) | 0.721 |
| Delay from onset to IVMP, median (IQR), days | 12.00  (5.00-16.50) | 12.00  (5.00-16.00) | 11.50  (4.25-17.00) | 0.762 |  |  | 10.00  (4.00-15.00) | 8.50  (4.00-15.00) | 11.50  (6.75-15.00) | 0.878 |
| Attack Visual acuity, logMAR, median (IQR) | 1.85  (1.00-2.70) | 1.85  (1.00-2.70) | 1.85  (1.21-2.92) | 0.622 |  |  | 1.85  (1.00-2.70) | 2.30  (1.18-2.78) | 1.85  (1.00-2.06) | 0.535 |
| Female, n (%) | 39 (90.7) | 30 (90.91) | 9 (90.00) | 1.000 |  |  | 21 (87.5) | 14 (87.50) | 7 (87.50) | 1.000 |
| Abbreviations: PSM = Propensity score matching; SD = standard deviation; IQR = inter-quartile range; PE = Plasma exchange; IA = Immunoadsorption; IVMP = Intravenous methylprednisolone therapy. | | | | | | | | | | |

| **Supplement Table 2.** Baseline Characteristics of other Clinical phenotype (except Optic neuritis) Groups Before and After 1:2 Matching for Predictive Variables. | | | | | | | | | | |
| --- | --- | --- | --- | --- | --- | --- | --- | --- | --- | --- |
|  | **Before PSM** | | | |  |  | **After PSM** | | | |
|  | **Total**  **(n = 244)** | **IVMP**  **(n = 222)** | **PE/IA+IVMP**  **(n = 22)** | **P** |  |  | **Total**  **(n = 66)** | **IVMP**  **(n = 44)** | **PE/IA+IVMP**  **(n = 22)** | **P** |
| Age, mean (SD), years | 45.69 (15.78) | 45.60 (15.94) | 46.64 (14.27) | 0.769 |  |  | 44.80 (16.25) | 43.89 (17.24) | 46.64 (14.27) | 0.521 |
| Delay from onset to IVMP, median (IQR), days | 15.00  (10.00-20.00) | 15.00  (10.00-20.00) | 11.50  (7.00-16.50) | 0.105 |  |  | 12.00  (8.00-16.75) | 12.50  (9.00-16.25) | 11.50  (7.00-16.50) | 0.662 |
| Attack EDSS, median (IQR) | 4.75  (3.50-7.00) | 4.50  (3.50-7.00) | 6.25  (4.50-8.38) | **0.023** |  |  | 6.50  (4.50-8.00) | 6.50  (4.50-8.00) | 6.25  (4.50-8.38) | 0.821 |
| Female, n (%) | 215 (88.11) | 195 (87.84) | 20 (90.91) | 0.937 |  |  | 60 (90.91) | 40 (90.91) | 20 (90.91) | 1.000 |
| Abbreviations: PSM = Propensity score matching; SD = standard deviation; IQR = inter-quartile range; PE = Plasma exchange; IA = Immunoadsorption; IVMP = Intravenous methylprednisolone therapy. | | | | | | | | | | |

| **Supplement Table 3.** Univariate regression analysis of AQP4-IgG titers at attack in EDSS improvement | | | |
| --- | --- | --- | --- |
| **Titers Grade** | **AQP4-IgG titers** | **Odds Ratio for EDSS improvement (95% CI)** | **P value** |
| **Grade 1** | **titers ≤ 1：10** | 1.000 (Reference) | - |
| **Grade 2** | **1：10 < titers ≤ 1：32** | 0.200 (0.011 - 3.661) | 0.278 |
| **Grade 3** | **1：32 < titers ≤ 1：100** | 0.800 (0.093 - 6.848) | 0.839 |
| **Grade 4** | **titers > 1：100** | 0.700 (0.090 - 5.432) | 0.733 |
| Abbreviations: EDSS = Expanded Disability Status Scale. | | | |
